# Typhoid Fever at Almont, Mich.

**Published:** 1853-02

**Authors:** 


*Typhoid Fever at Almont, Mich.*

OUR readers will find, in the Original Department of this number, a communication from Dr. BAILEY, containing the results of his observations and experience in the treatment of an epidemic fever, which prevailed in his neighborhood some years since.

We refer to this article for the purpose of calling the attention of the profession to the peculiar circumstances under which, in this instance, a disease, resembling in every respect typhoid fever, made its appearance.

The epidemic, of which the history is given by Dr. BAILEY, had its origin doubtless in the products resulting from the decomposition of vegetable matter; but whether or not the compounds resulting from the decay of bulbous roots, like the potato, in large heaps, would be the same, or similar to those generated during the disorganization of wood, leaves, and grass, scattered over a large surface, and therefore more fully exposed to the atmosphere, light, and heat, is, to our mind, a question of much interest and importance, especially when considered in connection with the question of identity of periodic and continued fevers.

Investigations upon this point have led us to the conclusion, that the decomposition of potatoes, in the manner and under the circumstances described by Dr. BAILEY, would give rise to highly nitrogenized compounds, unlike the hydro-carbonaceous products generated during the decay of vegetable matter, under circumstances favorable to the production of the so-called miasm of fever and ague districts. This fact, when taken in connection with others, such as the generation of typhoid fever on board of emigrant ships, in hospitals, and in ill-ventilated dwellings, where there comes to be an accumulation of nitrogenized effete matter, seems to indicate that the type of the disease may be determined by the presence or absence of nitrogenized gases.

We propose, at some future time, to examine this subject more at length; as we think it will furnish valuable indications of treatment. H.

